# Menstrual cycle and oral contraceptives influence cerebrovascular dynamics during hypercapnia

**DOI:** 10.14814/phy2.15373

**Published:** 2022-07-12

**Authors:** Tania J. Pereira, Sara Wasef, Ilana Ivry, Elnaz Assadpour, Baithat O. Adeyinka, Heather Edgell

**Affiliations:** ^1^ School of Kinesiology and Health Science York University Toronto Ontario Canada; ^2^ Muscle Health Research Centre York University Toronto Ontario Canada

**Keywords:** brain blood velocity, cerebral autoregulation, hypercapnia, oral contraceptives, transcranial doppler

## Abstract

Women experience fluctuating orthostatic intolerance during the menstrual cycle, suggesting sex hormones may influence cerebral blood flow. Young (aged 18–30) healthy women, either taking oral contraceptives (OC; *n* = 14) or not taking OC (NOC; *n* = 12), were administered hypercapnic gas (5%) for 5 min in the low hormone (LH; placebo pill) and high hormone (HH; active pill) menstrual phases. Hemodynamic and cerebrovascular variables were continuously measured. Cerebral blood velocity changes were monitored using transcranial doppler ultrasound of the middle cerebral artery to determine cerebrovascular reactivity. Cerebral autoregulation was assessed using steady‐state analysis (static cerebral autoregulation) and transfer function analysis (dynamic cerebral autoregulation; dCA). In response to hypercapnia, menstrual phase did not influence static cardiovascular or cerebrovascular responses (all *p* > 0.07); however, OC users had a greater increase of mean middle cerebral artery blood velocity compared to NOC (NOC‐LH 12 ± 6 cm/s vs. NOC‐HH 16 ± 9 cm/s; OC‐LH 18 ± 5 cm/s vs. OC‐HH 17 ± 11 cm/s; *p* = 0.048). In all women, hypercapnia improved high frequency (HF) and very low frequency (VLF) cerebral autoregulation (decreased nGain; *p* = 0.002 and <0.001, respectively), whereas low frequency (LF) Phase decreased in NOC‐HH (*p* = 0.001) and OC‐LH (*p* = 0.004). Therefore, endogenous sex hormones reduce LF dCA during hypercapnia in the HH menstrual phase. In contrast, pharmaceutical sex hormones (OC use) have no acute influence (HH menstrual phase) yet elicit a chronic attenuation of LF dCA (LH menstrual phase) during hypercapnia.

## Graphical abstract

The purpose of this study was to determine the influence of menstrual cycle and oral contraceptive use on the cerebral blood flow and autoregulatory response to hypercapnia. We found that oral contraceptive users had a greater blood flow velocity response to hypercapnia compared to non‐users. We also found that where endogenous sex hormones reduce low frequency dynamic cerebral autoregulation during hypercapnia in the high hormone menstrual phase, pharmaceutical sex hormones (oral contraceptive use) have no acute influence (high hormone menstrual phase) yet elicit a chronic attenuation of low frequency dynamic cerebral autoregulation (low hormone menstrual phase) during hypercapnia.

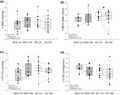

## 1 INTRODUCTION

Cerebral autoregulation is the process by which the cerebrovasculature responds to changes in perfusion pressure to ensure that cerebral blood flow (CBF) is maintained, in order to meet the high metabolic rate and waste production of the brain (Lassen & Christensen, 1976). Cerebral autoregulation can be assessed statically or dynamically; static cerebral autoregulation considers changes in pressure and flow over a longer time period, while dynamic cerebral autoregulation (dCA) involves immediate changes (Aaslid et al., 1989; Tiecks et al., 1995). There are four proposed mechanisms through which CBF is controlled; changes in vasomotor tone in response to pressure, activation of neural pathways to manipulate vasomotor tone, increased concentrations of metabolic by‐products (H+, pCO_2_, etc.), and the release of endothelial factors such as nitric oxide (Armstead, 2016). The cerebrovasculature is highly responsive to arterial CO_2_ levels, which means that vasodilation will occur during hypercapnia, and vasoconstriction will occur during hypocapnia to exponentially alter CBF (Battisti‐Charbonney et al., 2011; Hoiland et al., 2019; Meng & Gelb, 2015; Ogoh et al., 2008).

In healthy women, estrogen has been associated with reductions in resting cerebrovascular resistance during the late follicular menstrual cycle phase (i.e., high level of estrogen, no progesterone) compared to the early follicular phase (Krejza et al., 2003). However, Hazlett and Edgell (2018) observed no menstrual phase effects on the cerebrovascular response to CO_2_ when comparing the early follicular and luteal (i.e. high estrogen and progesterone) phases, suggesting that progesterone may counteract the effect of estrogen on the CO_2_ response. Notably, neither Peltonen et al. (2016) nor Hazlett and Edgell (2018) included women taking oral contraceptives (OC) and did not concurrently investigate indices of dCA. OC users have been shown to have lower resting levels of end‐tidal CO_2_ (ETCO_2_) (Abidi et al., 2017; Assadpour et al., 2020), which could have an influence on CBF parameters. Additionally, Abidi et al. (2017) observed that OC users did not concurrently increase cerebrovascular resistance in response to the observed hypocapnia. These results imply that OC users may have reduced cerebrovascular responsiveness to CO_2_ in response to hypocapnia. Furthermore, OC use is also associated with lower mean arterial pressure (MAP) and cerebral perfusion pressure during the active dose of the pill, suggesting that the use of exogenous cycling hormones may promote chronic peripheral vasodilation leading to lower blood pressure (Abidi et al., 2017). Indeed, the active dose of OC has been associated with increased lower limb blood flow (Minson et al., 2000a), reduced sympathetic baroreflex sensitivity (Minson et al., 2000a), and attenuated chemoreflex function (Usselman et al., 2013). Reduced sympathetic activity may impair dCA as evidenced by an increased magnitude of a change in CBF driven by blood pressure (i.e., Gain) during sympathetic blockade (Hamner et al., 2010).

This study aims to compare the effects of fluctuating endogenous and exogenous sex hormones on cerebrovascular CO_2_ reactivity and dCA. Abidi et al. (2017) observed a trend (*p* = 0.065) for greater cerebrovascular resistance with similar MAP in the high hormone (HH) phase of the menstrual cycle. Therefore, we hypothesized that women in the HH menstrual phase would have greater cerebrovascular resistance than women in the low hormone (LH) menstrual phase. Women are twice as likely to experience syncope than men, and syncope becomes more prevalent during puberty (i.e., when sex hormones exponentially increase) (Ganzeboom et al., 2003), suggesting that reduced dCA may be associated with the presence of sex hormones. Therefore, we hypothesize that in the presence of sex hormones in the HH menstrual phase, dCA will be reduced. Since OC users have lower ETCO_2_ (Abidi et al., 2017; Assadpour et al., 2020) and no change in the cerebrovascular resistance response (Abidi et al., 2017), we hypothesize that OC users would have enhanced CO_2_ reactivity compared to non‐users. Lastly, improved dCA has been observed during the second half of pregnancy compared to non‐pregnant individuals (i.e., pregnancy sex hormone concentrations are greater than across a menstrual cycle) (Janzarik et al., 2014; van Veen et al., 2016), suggesting that higher levels of sex hormones may have a positive effect on dCA. While pregnancy is also associated with severe hemodynamic changes, it represents a state of heightened levels of sex hormones, and previous research has shown that OC use has similar levels of sex hormone‐binding globulin which is reflective of total circulating sex hormone levels (van Kammen et al., 1975). This leads to our hypothesis that concentrations of sex hormones higher than typically seen through a natural cycle, such as those in OC active pills, may improve dCA.

## 2 METHODS

### 2.1 Participants

All participants in this study concurrently participated in Assadpour et al. (2020). Written informed consent was obtained prior to participation, which was approved by the Office of Research Ethics at York University. Young, healthy women (*n* = 26) between 18–35 years of age with a regular menstrual cycle (cycle length: 26–30 days) were recruited. Exclusion criteria included a history of hormonal or cardiovascular conditions and the inability to refrain from caffeine, fatty foods, and heavy exercise for 12 h prior to testing. We did not confirm the chronic smoking status of the participants; however, individuals who could not abstain from smoking 12 h prior to the testing were excluded from participation.

Women were separated into two groups, women not taking OC (NOC, *n* = 12) and women taking OC (OC, *n* = 14). OC participants reported using monophasic pills including Cyclen 28 (*n* = 3), Yaz (*n* = 2), Alesse (*n* = 3), Alysena (*n* = 4), and Marvelon (*n* = 2). Transcranial doppler ultrasound (TCD) measurements were unsuccessful in two participants (NOC = 1; OC = 1, Cyclen 28); therefore, those participants were removed from the analysis. Additionally, not all participants met the coherence threshold (0.63) for suitable transfer function analysis and thus, fewer participants were included in the analysis of dCA (see Table 3 for *n* values). Testing occurred during days 2–5 and 18–24 of the menstrual cycle for NOC participants (LH and HH menstrual phases, respectively), while OC participants were tested during the placebo week (LH menstrual phase) and the final OC pill week (HH menstrual phase). Self‐report was used to determine menstrual cycle phases. Progesterone production in the HH menstrual phase in NOC, and thus ovulation, was confirmed using a urine progesterone test (>5 μg/ml; Progesterone [PDG] urine test, Easy@Home). Testing was randomized to reduce any ordering effects between LH or HH menstrual phase testing sessions, and researchers were not blinded to OC use, nor which phase was being tested.

Anthropometrics (height, body mass) were measured using a standard stadiometer and used to calculate body mass index as body mass/height^2^. Information about anthropometrics and self‐reported physical activity were used to obtain an index of maximal oxygen consumption (VO_2_ max; ml kg^−1^ min^−1^), calculated with the Ainsworth Equation (1992).

### 2.2 Hypercapnia protocol

Participants laid supine for 5 min while breathing room air to establish a baseline. After baseline, participants started breathing a hypercapnic gas mixture (5% CO_2_, 21% O_2_, balance nitrogen) for 5 min, administered via a free‐breathing Douglas bag system with no set flow rate.

### 2.3 Measurements

All measurements were conducted in the supine posture, and data was acquired at a rate of 1000 Hz through PowerLab (16/35, ADInstruments) and LabChart Pro (Version 8.1.9, ADinstruments) software. Heart rate was continuously measured using a single‐lead electrocardiogram (BioAMP, ADInstruments). Blood pressure was determined by beat‐to‐beat finger plethysmography on the left middle finger (BMEye Nexfin) and was calibrated to an automated measurement using LabChart (BP Tru, BPM 200). ETCO_2_ was measured using a CO_2_ gas analyzer (Model 17630, Vacumed).

Right middle cerebral artery blood velocity (MCA_V_) was measured using a TCD system (Multigon Industries Inc.). A 2‐MHz TCD probe was placed on the temporal window above the zygomatic arch and held by an adjustable headband (Marc600 Headframe, Spencer Industries). MCA_V_ is described as mean (MCA_Vmean_), diastolic (MCA_Vmin_), and systolic (MCA_Vmax_). The ratio of the change in MCA_V_ to the change in ETCO_2_ was calculated. Cerebrovascular resistance index (CVRi) was calculated as MAP divided by MCA_Vmean_. Resistance index (RI) was calculated as the difference between MCA_Vmax_ and MCA_Vmin_, divided by MCA_Vmax_. Pulsatility index (PI) was calculated as the difference between MCA_Vmax_ and MCA_Vmin_, divided by the MCA_Vmean_. Resistance area product (RAP) was calculated as the difference between MAP and diastolic blood pressure divided by the difference between MCA_Vmean_ and MCA_Vmin_. Critical closing pressure (CrCP) was calculated as MAP minus the product of RAP and MCA_Vmean_.

Transfer function analysis was used to quantify dCA, according to the Claassen et al. (2016) white paper. Briefly, MCA_V_ and blood pressure were continuously obtained throughout each cardiac cycle, then were resampled at 4 Hz using Ensemble‐R software (Ensemble‐R, Elucimed Ltd.). The data set was divided into 4 windows, which were passed through a Hann (cosine‐bell) data window with an overlap of 50%. A fast Fourier transform was used to transform the signals into frequencies, which are binned into specific bands: high frequency (HF; 0.2–0.5 Hz), low frequency (LF; 0.07–0.2), and very low frequency (VLF; 0.02–0.07 Hz). Spectral analysis provides power spectra for MCA_Vmean_ and cerebral perfusion pressure (MAP_MCAv_), representing the magnitude of influence of similar frequency components on each variable. Cross‐spectral analysis is applied to the power of MCA_Vmean_ and MAP_MCAv_ to create the transfer function, which describes how pressure fluctuations translate to changes in blood velocity via the amplitude (Gain) and timing (Phase) of the relationship. Gain can be normalized (nGain) by expressing velocity as a percentage of the mean signal to account for between‐group differences. A large Gain implies that changes in pressure lead to greater fluctuations in velocity, and a smaller Phase implies that pressure fluctuations are more quickly transmitted to velocity, indicating poor dCA (Claassen et al., 2016). Coherence (ranges from 0–1), an additional parameter calculated by transfer function analysis, describes the reliability of the relationship between Phase and Gain. Each variable of our dCA assessments (MCA_Vmean_ power, MAP_MCAv_ power, Gain, nGain, Phase, and Coherence) are determined across each frequency band; HF, LF, and VLF. Ensemble‐R software quantifies parameters that meet a linearity threshold based on a 95% confidence interval (default coherence threshold: 0.63). Phase wrap‐around was present in the HF and LF bands, and those values were removed from the analysis (Claassen et al., 2016).

### 2.4 Data analysis

For steady‐state assessments, 1 min averages were determined to compare baseline to hypercapnia, and only the last minute of each was used for statistical analysis. Changes were calculated by subtracting the baseline values from the last minute of hypercapnia administration. Static participant characteristics (i.e., age and height) were compared between OC and NOC using independent *t*‐tests, while other characteristics (i.e., body mass, body mass index, exercise frequency, and estimated VO_2_ max) were compared using a 2‐way repeated‐measures ANOVA (OC use and menstrual phase as factors). Changes in cardiovascular (heart rate, MAP), static cerebrovascular (MCA_V_, resistance indices), and ventilatory (ETCO_2_) responses to hypercapnia administration were compared using a 2‐way repeated‐measures ANOVA (OC use and menstrual phase as factors). A 3‐way repeated‐measures ANOVA was used to compare transfer function analysis variables, with OC use as a between‐participant factor and menstrual phase and CO_2_ (baseline and hypercapnia) as within‐participant factors.

Statistical analyses were performed using SPSS Statistics software (Version 25, IBM) and in consultation with a biostatistician. Significance was set a priori to *p* < 0.05, and all data in Tables are presented as mean ± SD while data in Figures are presented as median with 25th and 75th percentiles. Each variable was assessed for normality, using the Shapiro–Wilk test for small sample size and a visual analysis of boxplots. Variables that violated the assumption of normality and were skewed (outside the range of −1 to 1) had a gamma distribution applied and were log‐transformed. Post‐hoc analysis was conducted using estimated marginal means and pairwise comparisons, with a Sidak correction to account for multiple comparisons. Cohen's D (*d*) was used as a marker of effect size for all significant differences and was calculated as the mean difference between groups (grouped by hormone phase, time point, and/or OC use depending on significant interactions), then divided by the pooled standard deviation.

## 3 RESULTS

### 3.1 Participant characteristics and cerebrovascular hemodynamics

**FIGURE 1:**
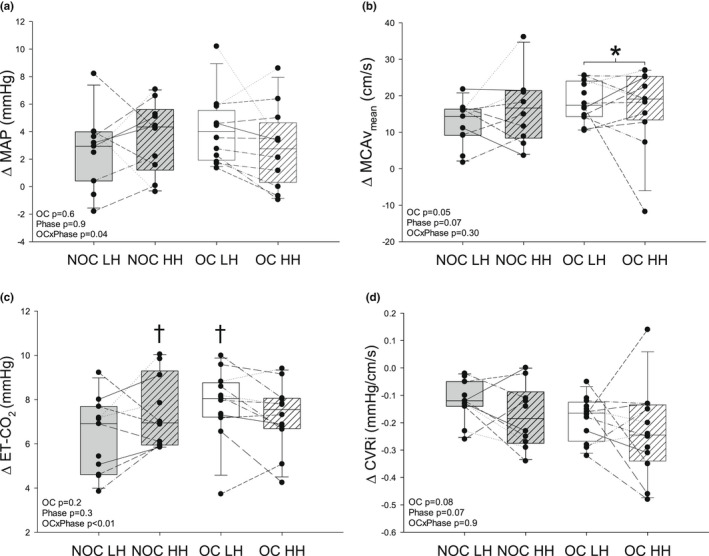
Change in cerebral mean arterial pressure (MAP; a), mean cerebral artery blood velocity (MCA_Vmean_; b), end‐tidal carbon dioxide (ETCO_2_; c) and cerebrovascular resistance index (CVRi; d) in response to 5 min of hypercapnia in women taking oral contraceptives (OC; white bar) and not taking oral contraceptives (NOC; gray bar) during the low hormone (LH) and high hormone (HH) phases of their menstrual cycle. The centerline of each box represents the median response, while the upper and lower border of the box represents the 25th and 75th percentile. *Significantly different than NOC users; ^†^A significant difference from NOC‐LH.

Participant characteristics were not different between groups and/or menstrual phases compared by age, height, body mass, body mass index, resting systolic blood pressure, resting diastolic blood pressure, or predicted VO_2_ max (all *p* > 0.10; Table 1). There was no significant difference in the heart rate response to hypercapnia between menstrual phases or OC use (all *p* > 0.1; Table 2). Similarly, many steady‐state cerebrovascular responses to hypercapnia were not different between menstrual phases or with OC use: MCA_Vmin_ (all *p* > 0.06; Table 2), MCA_Vmax_ (all *p* > 0.08; Table 2), RAP (all *p* > 0.07; Table 2), CrCP (all *p* > 0.2; Table 2), RI (all *p* > 0.1; Table 2), and PI (all *p* > 0.2; Table 2). There was a significant interaction between menstrual phase and OC use on the MAP response to hypercapnia (*p* = 0.036; Figure 1a); however, post hoc analysis revealed that there were no significant differences (all *p* > 0.05). Regardless of menstrual phase (*p* = 0.07), OC users had a significantly greater MCA_Vmean_ response to hypercapnia (*p* = 0.048; *d* = 0.43; Figure 1b). The ETCO_2_ response to administration of hypercapnia was lower in NOC‐LH compared to both NOC‐HH (*p* = 0.039; *d* = 0.66) and OC‐LH (*p* = 0.03; *d* = 0.87; Figure 1c). Regardless of menstrual phase, OC users had a higher slope between the change in MCA_Vmean_ over the change in ETCO_2_ (NOC‐LH: 1.85 ± 0.68, NOC‐HH: 2.14 ± 1.09, OC‐LH: 2.38 ± 0.50, OC‐HH: 2.41 ± 1.49; Main effect of OC *p* = 0.03; OC vs. NOC *d* = 0.41). There were no significant effects of the menstrual cycle (*p* = 0.07) or OC use (*p* = 0.08) on the change in CVRi during hypercapnia (Figure 1d).

**TABLE 1:** Anthropometrics and predicted fitness for OC users, compared to NOC

	NOC		OC		Comparisons (*p*‐value)		
	LH	HH	LH	HH	Main effects		Interactions
					OC	Phase	Phase*OC
Age (years)	22 ± 4		22 ± 4		0.78	—	—
Height (cm)	163 ± 5		163 ± 7		0.75	—	—
Body mass (kg)	64 ± 10	64 ± 10	67 ± 12	67 ± 12	0.41	0.85	0.48
Body mass index (kg m^−2^)	24 ± 3	24 ± 3	25 ± 4	25 ± 4	0.39	0.56	0.24
Systolic blood pressure (mmHg)	106 ± 9	108 ± 15	109 ± 8	108 ± 8	0.74	0.72	0.56
Diastolic blood pressure (mmHg)	68 ± 8	68 ± 11	72 ± 7	73 ± 6	0.10	0.87	0.87
Predicted VO_2_ max (ml kg^−1^ min^−1^)	37 ± 4	37 ± 3	36 ± 2	36 ± 2	0.50	0.79	0.78

*Note*: All values are mean ± SD. No significant differences between OC and non‐OC users (*p* > 0.05).

Abbreviations: HH, high hormone; LH, low hormon; NOC, no oral contraceptive; OC, oral contraceptive; VO_2_ max, maximum oxygen consumption.

**TABLE 2:** Changes in cardiovascular and cerebrovascular variables in response to hypercapnia

	NOC		OC		Comparisons (*p*‐value)		
	LH	HH	LH	HH	Main effects		Interactions
	∆	∆	∆	∆	OC	Phase	Phase*OC
HR (bpm)	2 ± 3	5 ± 4	2 ± 4	3 ± 4	0.57	0.29	0.19
MCAvmin (cm/s)	11 ± 6	13 ± 9	15 ± 5	13 ± 10	0.23	0.11	0.06
MCAvmax (cm/s)	11 ± 5	15 ± 9	17 ± 7	18 ± 13	0.08	0.25	0.65
CrCP (mmHg)	−2.19 ± 2.38	−2.90 ± 5.94	−2.61 ± 2.5	−0.59 ± 4.97	0.49	0.54	0.21
RAP (mmHg/cm/s)	−0.01 ± 0.04	−0.02 ± 0.06	−0.03 + 0.03	−0.06 + 0.06	0.07	0.15	0.42
RI	−0.04 ± 0.03	−0.06 ± 0.05	−0.06 ± 0.02	−0.04 ± 0.05	0.89	0.73	0.13
PI	−0.11 ± 0.08	−0.16 ± 0.12	−0.15 ± 0.07	−0.13 ± 0.15	0.82	0.72	0.24

*Note*: All values are mean ± SD.

Abbreviations: CrCP, critical closing pressure; HH, high hormone; HR, heart rate; LH, low hormone; MCA_Vmax,_ maximum middle cerebral artery velocity; MCA_Vmin_, minimum middle cerebral artery velocity; NOC, no oral contraceptive; OC, oral contraceptive; PI, pulsatility index; RAP, resistance area product; RI, resistance index.

**TABLE 3:** Transfer function analysis of cerebrovascular response to hypercapnia

	NOC				OC				Comparisons (*p*‐value)						
	LH		HH		LH		HH		Main effects			Interactions			
Frequency Range	Baseline *n* = 9	CO_2_ *n* = 9	Baseline *n* = 10	CO_2_ *n* = 11	Baseline *n* = 10	CO_2_ *n* = 10	Baseline *n* = 12	CO_2_ *n* = 11	OC	Phase	CO_2_	OC Phase	OC CO_2_	Phase CO_2_	OC Phase CO_2_
HF															
MCA_Vmean_ power, cm^2^/s^2^	5.71 ± 4.04	6.30 ± 4.84	6.95 ± 3.76	10.73 ± 8.04^a^ ^,^ ^c^	6.99 ± 6.58	7.61 ± 4.83	6.88 ± 6.41	10.33 ± 7.71^a^ ^,^ ^c^	0.731	—	—	0.487	0.921	**0.019**	0.959
MAP_MCAv_ power, mmHg^2^	2.11 ± 3.09	2.47 ± 4.01	2.77 ± 1.87	3.50 ± 2.34	1.35 ± 0.97	1.53 ± 1.21	1.72 ± 1.54	2.79 ± 2.52	0.225	0.170	**0.003**	0.834	0.516	0.094	0.282
Coh, au	0.52 ± 0.20	0.57 ± 0.16	0.53 ± 0.17	0.60 ± 0.12	0.53 ± 0.13	0.59 ± 0.19	0.56 ± 0.15	0.57 ± 0.16	0.867	0.690	**0.024**	0.852	0.721	0.764	0.492
Gain, cm/s/mmHg	1.89 ± 0.74	1.83 ± 0.82	1.53 ± 0.40	1.66 ± 0.70	2.06 ± 0.93	2.40 ± 1.40	2.02 ± 0.89	2.23 ± 1.41	0.141	0.325	0.055	0.601	0.190	0.689	0.285
nGain, %/mmHg	2.30 ± 1.04	1.97 ± 0.93	2.13 ± 0.79	1.95 ± 0.91	2.52 ± 1.01	2.46 ± 1.21	2.65 ± 0.78	2.34 ± 1.05	0.201	0.842	**0.002**	0.834	0.593	0.753	0.232
Phase, radians	0.42 ± 0.56	0.32 ± 0.22	0.34 ± 0.33	0.17 ± 0.13	0.27 ± 0.17	0.11 ± 0.15	0.25 ± 0.22	0.16 ± 0.13	0.122	0.662	0.060	0.356	0.778	0.990	0.506
LF															
MCA_Vmean_ power, cm^2^/s^2^	7.27 ± 4.97	6.89 ± 3.05	7.67 ± 4.99	8.43 ± 5.93	7.48 ± 4.06	6.99 ± 4.53	6.50 ± 3.24	9.46 ± 8.53	0.996	0.446	0.355	0.866	0.479	0.136	0.457
MAP_MCAv_ power, mmHg^2^	5.97 ± 9.59	4.57 ± 5.13	3.65 ± 2.21	4.55 ± 4.39	2.95 ± 2.71	4.06 ± 5.34	2.68 ± 2.41	3.51 ± 3.38	0.296	0.506	0.219	0.814	0.151	0.365	0.267
Coh, au	0.55 ± 0.18	0.60 ± 0.24^b^	0.61 ± 0.16	0.61 ± 0.22	0.51 ± 0.17	0.64 ± 0.15^b^	0.53 ± 0.18	0.52 ± 0.18	0.504	**—**	**—**	0.256	0.431	**0.004**	0.171
Gain, cm/s/mmHg	1.36 ± 0.61	1.45 ± 0.58	1.37 ± 0.43	1.50 ± 0.54	1.38 ± 0.43	1.63 ± 0.69	1.42 ± 0.48	1.78 ± 0.92	0.344	0.737	**0.027**	0.869	0.322	0.666	0.906
nGain, %/mmHg	1.70 ± 0.99	1.58 ± 0.79	1.85 ± 0.73	1.72 ± 0.66	1.87 ± 0.62	1.70 ± 0.59	1.84 ± 0.52	1.87 ± 0.66	0.542	0.602	0.256	0.857	0.769	0.670	0.618
Phase, radians	0.52 ± 0.20	0.44 ± 0.24^d^	0.60 ± 0.40	0.28 ± 0.18^a^	0.58 ± 0.33	0.34 ± 0.23^b^	0.66 ± 0.38	0.68 ± 0.33^c^ ^,^ ^e^	**—**	**—**	**—**	**—**	**—**	**—**	**0.025**
VLF															
MCA_Vmean_ power, cm^2^/s^2^	18.23 ± 22.15	6.46 ± 3.30^b^	18.77 ± 15.27	7.39 ± 6.55^a^	25.47 ± 29.13	7.99 ± 6.17^b^	14.56 ± 12.61	11.49 ± 20.17^a^	0.554	**—**	**—**	0.677	0.440	**0.050**	0.120
MAP_MCAv_ power, mmHg^2^	9.52 ± 15.71	6.04 ± 7.18	5.72 ± 6.50	3.51 ± 3.12	4.01 ± 2.46	3.08 ± 2.00	3.41 ± 2.81	4.23 ± 4.26	0.129	0.427	**0.035**	0.284	0.057	0.302	0.239
Coh, au	0.43 ± 0.18	0.40 ± 0.17	0.46 ± 0.15	0.48 ± 0.14	0.36 ± 0.12	0.43 ± 0.15	0.35 ± 0.13	0.41 ± 0.13	0.231	0.444	0.273	0.180	0.169	0.727	0.538
Gain, cm/s/mmHg	2.29 ± 1.28	1.56 ± 0.78	1.64 ± 0.69	1.26 ± 0.55	1.44 ± 0.77	1.27 ± 0.63	2.18 ± 1.50	1.69 ± 0.82	**—**	**—**	**0.001**	**0.040**	0.375	0.952	0.337
nGain, %/mmHg	2.90 ± 1.92	1.67 ± 0.93	2.21 ± 1.19	1.48 ± 0.74	1.72 ± 1.03	1.43 ± 0.67	2.66 ± 1.95	1.80 ± 0.70	0.670	0.673	**<0.001**	0.099	0.248	0.855	0.213
Phase, radians	0.80 ± 0.54	0.93 ± 0.45	0.98 ± 0.72	0.56 ± 0.52	0.56 ± 0.41	0.34 ± 0.69	0.86 ± 0.75	0.95 ± 0.44	0.383	0.211	0.369	0.054	0.724	0.621	0.057

*Note*: All values are mean ± SD. Significance is represented by bold text for main effects and interactions (*p* < 0.05).

Abbreviations: Coh, Coherence; HF, high frequency (0.2–0.4 Hz); HH, high hormone; LF, low frequency (0.07–0.2 Hz); LH, low hormone; MAP_MCAv_, mean arterial pressure height‐corrected to the middle cerebral artery; MCAv_mean_, middle cerebral artery mean blood velocity; nGain, normalized Gain; NOC, no oral contraceptive; OC, oral contraceptive; VLF, very low frequency (0.02–0.07 Hz).

^a^ Significantly different than baseline in HH phase.

^b^ Significantly different than baseline in LH phase.

^c^ Higher than CO_2_ in LH phase.

^d^ Higher than CO_2_ in HH phase.

^e^ Different than NOC at this timepoint.

### 3.2 Dynamic cerebral autoregulation

Regardless of menstrual cycle or OC use, hypercapnia increased HF power of MAP_MCAv_ (*p* = 0.003; *d* = 0.26), increased HF Coherence (*p* = 0.024; *d* = 0.34), and decreased HF nGain (*p* = 0.002; *d* = 0.21). There was a significant interaction between menstrual phase and hypercapnia for the HF power of MCA_Vmean_ (*p* = 0.019), where hypercapnia increased the HF power of MCA_Vmean_ in the HH menstrual phase only (*p* < 0.001; *d* = 0.02). During hypercapnia, the HF power of MCA_Vmean_ was significantly higher in the HH than LH menstrual phase (*p* = 0.036; *d* = 0.55).

Hypercapnia increased LF Gain in all women regardless of phase (*p* = 0.027; *d* = 0.32) and increased LF Coherence only in the LH menstrual phase (*p* = 0.001; *d* = 0.51). There was a significant interaction between OC, menstrual phase, and hypercapnia in LF Phase (*p* = 0.025). In response to hypercapnia, LF Phase decreased significantly in the NOC group during the HH menstrual phase (*p* = 0.001; *d* = 1.05), leading to LF Phase being higher during hypercapnia in the LH than in the HH menstrual phase (*p* = 0.005; *d* = 0.66). In contrast, due to hypercapnia, LF Phase decreased significantly in the OC group during the LH menstrual phase (*p* < 0.004; *d* = 0.76), leading to the LF Phase being larger in the HH menstrual phase compared to the LH menstrual phase during hypercapnia (*p* = 0.012, *d* = 0.85). In the HH menstrual phase during hypercapnia, OC users had a larger LF Phase than NOC (*p* < 0.001, *d* = 0.78).

In all women, hypercapnia decreased VLF power of MAP_MCAv_ (*p* = 0.035; *d* = 0.20), VLF Gain (*p* = 0.001; *d* = 0.53), and VLF nGain (*p* < 0.001; *d* = 0.72), regardless of menstrual phase. There was a significant interaction between menstrual phase and hypercapnia for VLF power of MCA_Vmean_ (*p* = 0.050); although after considering post‐hoc comparisons, there was no difference in the hypercapnic response between menstrual phases (all *p* > 0.255). In response to hypercapnia, the VLF power of MCA_Vmean_ decreased in the LH menstrual phase (*p* = 0.002; *d* = 0.76) and HH menstrual phase (*p* = 0.025, *d* = 0.49). There was a significant interaction between menstrual phase and OC use for VLF Gain (*p* = 0.040), but post‐hoc analysis revealed that there were no significant differences between groups (all *p* > 0.122).

## 4 DISCUSSION

The aim of this study was to describe the cerebrovascular and dCA response to hypercapnia during fluctuating phases of endogenous and exogenous sex hormones. In response to hypercapnia, neither the cardiovascular nor the cerebrovascular responses were influenced by menstrual phase, yet OC users had a greater increase in MCA_Vmean_. Hypercapnia significantly decreased VLF and HF nGain in all women and menstrual phases. NOC users in the HH menstrual phase had decreased LF Phase in response to hypercapnia, whereas OC users experienced LF Phase decline in the LH menstrual phase in response to hypercapnia administration. Therefore, our key findings are that (1) there are minimal effects of menstrual phase or OC on hemodynamic or cerebrovascular resistance responses to hypercapnia, (2) all women exhibit enhanced HF and VLF dCA via dampening of blood pressure fluctuations (nGain) in hypercapnia, and (3) while naturally cycling women have reduced timing (Phase) of LF changes in blood pressure translating to changes in CBF during hypercapnia in the HH menstrual phase, OC users exhibit the same reduction in the LH menstrual phase.

### 4.1 Cardiovascular, ventilatory, and cerebrovascular response to hypercapnia

In the parallel study, Assadpour et al. (2020) found no influence of menstrual cycle nor OC use on resting cardiorespiratory variables. Similarly, the present study found no effects of menstrual phase or OC use on the hemodynamic responses to hypercapnia. Usselman et al. (2013) also found that menstrual phase in OC users did not influence hemodynamic responses to hypoxic hypercapnia via apnea. ETCO_2_ during 5% CO_2_ administration was lower in NOC‐LH compared to both NOC‐HH and OC‐LH. However, it is important to note that while minor differences in ventilation and/or pulmonary perfusion could play a small role, the median values between groups differed by <1 mmHg. This small difference in ETCO_2_ is unlikely to influence either cerebrovascular resistance or autoregulation. Indeed, a 7.5 mmHg increase in ETCO_2_ during hypercapnia administration does not elicit a change in middle cerebral artery diameter (Verbree et al., 2014), suggesting that the present difference is not likely to influence diameter or perhaps dCA.

Contrary to our hypotheses, OC users had a similar MAP response to hypercapnia in the HH menstrual phase compared to the LH menstrual phase, yet the MCA_V_ response to hypercapnia was greater than NOC regardless of pill phase. Since there was a significantly greater MCA_V_ response but no change in MAP response in OC users, we expected to see a reduction in cerebrovascular resistance (i.e., CVRi, RAP, CrCP, RI, and PI), yet this was not statistically significant. A greater sample number and statistical power or controlling for OC pill generation could have decreased variability and provided evidence for changes in resistance indices. However, OC use did not influence cerebrovascular resistance indices throughout a supine‐sit‐stand model, which includes both hypocapnia and baroreflex activation (Abidi et al., 2017), nor does OC pill generation influence peripheral artery vasodilatory capacity (Shenouda et al., 2018). Similarly, menstrual phase did not influence indices of cerebrovascular resistance in response to hypercapnia, corresponding to previous work in our lab, which found that there is no influence of the menstrual cycle on cerebrovascular resistance indices during upright posture (Abidi et al., 2017; Hazlett & Edgell, 2018).

### 4.2 Dynamic cerebral autoregulation

In all women during hypercapnia, the HF power of MAP control, HF Coherence, and the LF Gain (but not LF nGain) increased, while HF nGain, VLF power of MAP control, VLF Gain, and VLF nGain decreased. These results suggest that the HF component of MAP control is elevated while the VLF component is suppressed during hypercapnia. Furthermore, the HF and VLF components (nGain) of dCA are improved during hypercapnia (i.e., increased buffering of blood pressure fluctuations). Interestingly, Ainslie et al. (2008) observed no change in LF, HF, or VLF Gain, Coherence, MAP variability, or MCA_V_ variability during 4% hypercapnia in young recreationally active men. This may suggest that men are less likely to experience dCA dysregulation during hypercapnia compared to women. High levels of aerobic fitness are associated with both a reduced ability to dampen rapid or large changes in blood pressure (Labrecque et al., 2017, 2019) and increased cerebrovascular CO_2_ reactivity (Smith et al., 2021). Groups in the current study did not differ by fitness; however, this highlights the importance of considering fitness in investigations of CBF and cerebrovasculature.

The HF power of the MCA_V_ significantly increased during hypercapnia only in the HH menstrual phase, whereas LF Coherence increased during hypercapnia only in the LH menstrual phase, regardless of OC use. The exact physiological mechanisms for HF and LF control of CBF are unclear; however, the presence of estrogen and progesterone appear to augment the HF control while reducing the LF control in hypercapnia. Schmetterer et al. (1997) observed that MCA_Vmean_ increases during hypercapnia, which was blunted by infusion of a nitric oxide synthase inhibitor, suggesting that nitric oxide plays a key role in the hypercapnic vasodilatory response. Indeed, since estrogen is known to upregulate endothelial nitric oxide synthase (Gavin et al., 2009), an enhanced release of nitric oxide in the HH menstrual phase during hypercapnia could be responsible for the augmentation of HF control of CBF.

Coherence is based on the assumption of a linear relationship between MCA_V_ and blood pressure. We observed that hypercapnia increases this relationship in the LF range when estrogen and progesterone levels are reduced; however, the relationship does not increase in the presence of high levels of these hormones. Since resting neurovascular transduction of sympathetic input is enhanced in the absence of estrogen and progesterone (Usselman et al., 2015), we hypothesize that the increased LF Coherence during hypercapnia in the LH menstrual phase could be due to enhanced sympathetic output during hypercapnia, but the lack of change in LF Coherence during hypercapnia in the HH menstrual phase is due to impaired vascular transduction of this increased sympathetic activity. Korad et al. (2022) observed a negative relationship between estradiol concentration and Rate of Regulation (i.e., the rate of change in CVRi related to the change in blood pressure) during repeated squat maneuvers, suggesting that sex hormones are related to autoregulation. Since forced blood pressure oscillations (e.g., repeated squats) have been shown to increase coherence compared to spontaneous oscillations (Smirl et al., 2015), this may provide stronger evidence of diminished cerebral autoregulation during high levels of sex hormones. Future research should investigate the effects of OC use on forced blood pressure oscillations to improve the interpretation of the linear relationship between MCA_v_ and blood pressure.

Importantly, NOC users had a reduction of LF dCA during hypercapnia in the presence of estrogen and progesterone, while OC users had a reduction of LF dCA during hypercapnia in the absence of estrogen and progesterone analogs. Therefore, the reduction of LF dCA during hypercapnia observed in the presence of natural estrogen/progesterone is not seen in the presence of pharmaceutical estrogen/progesterone, yet the chronic use of OC (as indicated by the placebo pill phase of OC) also causes a reduction of LF dCA during hypercapnia. In naturally cycling women, enhanced sympathetic baroreflex sensitivity and an increased blood pressure response to hypercapnia exists in the HH menstrual phase compared to the LH menstrual phase (Edwards et al., 1996; Minson et al., 2000b). Furthermore, sympathetic baroreflex sensitivity is negatively associated with dCA, such that less effective dCA is associated with increased sympathetic baroreflex sensitivity and vice versa (Nasr et al., 2014; Tzeng et al., 2010; Witter et al., 2017). Therefore, an enhanced sympathetic influence during hypercapnia in the HH menstrual phase of naturally cycling women may be responsible for the reduced LF dCA observed in the current study. Interestingly, it has also been observed that OC users have greater sympathetic baroreflex sensitivity and increased sympathoexcitation in response to severe chemoreflex activation during the LH menstrual phase (Minson et al., 2000a; Usselman et al., 2013). Along with our observations, these studies again suggest a relationship between increased sympathetic influence and reduced LF dCA in OC users during hypercapnia in the LH menstrual phase.

### 4.3 Perspectives

Although the human brain represents only 2% of the average human's total body mass (Williams & Leggett, 1989), it requires roughly 20% of total body oxygen at rest (Rink & Khanna, 2011). This high demand for oxygenated blood means that the brain is susceptible to mismatches in perfusion and that decrements in the effectiveness of dCA can result in transient ischemia. While dCA was not different during baseline, the impact of hypercapnia might be particularly relevant to exercise, and a mismatch between perfusion pressure and flow could have detrimental effects for exercising women. Generally, dCA has been shown to be preserved in response to exercise of varying modalities and intensities (Brys et al., 2003; Herholz et al., 1987; Ogoh et al., 2010; Tsukamoto et al., 2019). However, more exhaustive aerobic or resistance exercise is associated with greater dCA dysregulation (Koch et al., 2005; Ogoh et al., 2005), suggesting that exercise can induce dCA dysregulation in an intensity‐dependant manner. However, a majority of these studies are conducted solely on men (Herholz et al., 1987; Ogoh et al., 2005; Tsukamoto et al., 2019), included insufficient female populations for statistical comparisons (Ogoh et al., 2010), or did not consider sex‐related differences (Brys et al., 2003; Koch et al., 2005). More exercise intervention studies that investigate women through the menstrual cycle while controlling for OC use are warranted.

### 4.4 Limitations

A limitation of the current study is that the analysis of OC was not separated by pill generation due to small sample sizes. It is possible that the type of progestin and formulation with estradiol could potentially affect physiological responses. Differences between OC generations have previously been observed by Shenouda et al. (2018), where a negative relationship between the duration of OC use and flow‐mediated dilation was observed in second‐generation OC only. This suggests that different pill formulations may lead to different vascular responses. Future studies should enlarge sample sizes to separate OC generations for comparison. The current study did not quantify estrogen or progesterone levels; however, urine samples from the HH phase trial were tested for the presence of progesterone to confirm ovulation. If sufficient progesterone was not present (<5 μg/ml), participants were asked to return to the lab during their next cycle to ensure that an appropriate level of progesterone was present. Finally, the present observations are cross‐sectional comparisons, which is a great first step to determining the effects of OC on autonomic functioning yet does not elucidate the longitudinal response within an individual. Longitudinal studies that observe women prior to starting their pill regimen are required to remove any baseline bias that could have led to the present observations. TCD ultrasound measures MCA_V_ to estimate CBF and is based on the assumption that the cerebrovascular diameter remains constant. While cerebrovascular diameter stability has been demonstrated for changes in ETCO_2_ smaller than 7.5 mmHg (Verbree et al., 2014), the present study cannot confirm if the cerebrovascular diameter was maintained. Furthermore, transfer function analysis was used to quantify autoregulation on spontaneous changes in blood pressure, which is related to lower Coherence (i.e., poorer signal‐to‐noise ratio) (Smirl et al., 2015), and reproducibility (i.e., within‐day) (Burma et al., 2020; Sanders et al., 2019). Methodologies regarding cerebrovascular CO_2_ reactivity have also been highly debated. The duration of the CO_2_ stimulus and steady‐state timepoints used for analysis can influence outcome variables (Burley et al., 2020). Finally, these observations are limited to the present participant pool (i.e., healthy menstruating women) and cannot be generalized to other areas of cerebral circulation (Skow et al., 2013).

## 5 CONCLUSIONS

In response to hypercapnia, while OC users had a greater increase in MCA_Vmean_ compared to NOC, neither the hemodynamic nor the cerebrovascular resistance responses were influenced by OC use or menstrual phase. We also observed that MAP control becomes more reliant on HF and less reliant on VLF control during hypercapnia. Additionally, VLF control of MCA_V_ decreased significantly during hypercapnia in both phases, yet the response did not differ throughout the menstrual cycle. Comparatively, HF MCA_V_ control is menstrual phase dependent in hypercapnia, where hypercapnia increases HF power in the presence of estrogen and progesterone analogs. In women, exposure to CO_2_ generally led to better autoregulation (HF and VLF nGain), although some aspects of dCA were dependent on menstrual phase and OC use (Coherence or Phase). Importantly, we found that LF dCA was reduced during hypercapnia in the HH menstrual phase of NOC, yet this reduction exists in the LH menstrual phase of OC users. This suggests that the response to hypercapnia in the presence of endogenous estrogen and progesterone is reduced LF dCA; however, this is not observed in the presence of pharmaceutical hormones. Furthermore, the chronic use of pharmaceutical hormones (as evidenced by changes during the placebo pill) elicits reduced LF dCA during hypercapnia. The current study highlights the importance of considering the influence of an individual's hormonal milieu.

## AUTHOR CONTRIBUTIONS

Tania J. Pereira, Sara Wasef, Elnaz Assadpour, Ilana Ivry, Baithat O. Adeyinka, and Heather Edgell made a substantial contribution to the concept and design; Tania J. Pereira, Sara Wasef, Elnaz Assadpour, Ilana Ivry, Baithat O. Adeyinka, and Heather Edgell were responsible for data acquisition or analysis and interpretation of data; Tania J. Pereira and Heather Edgell drafted the article or revised it critically for important intellectual content; Tania J. Pereira, Sara Wasef, Elnaz Assadpour, Ilana Ivry, Baithat O. Adeyinka, and Heather Edgell approved the version to be published.

## FUNDING INFORMATION

The Natural Sciences and Engineering Research Council of Canada, the Canadian Foundation for Innovation, and the Ontario Research Fund provided funding for this study.

## CONFLICT OF INTEREST

The authors have no conflicts to declare.
